# Recommendations from people who use drugs in Philadelphia, PA about structuring point-of-care drug checking

**DOI:** 10.1186/s12954-024-00937-8

**Published:** 2024-01-30

**Authors:** Megan K. Reed, Elias Borne, Tracy Esteves Camacho, Morgan Kelly, Kristin L. Rising

**Affiliations:** 1 Department of Emergency Medicine, Sidney Kimmel Medical College, Thomas Jefferson University, 1015 Walnut Street, Curtis Building, Suite 704, Philadelphia, PA 19107 USA; 2 Center for Connected Care, Sidney Kimmel Medical College, Thomas Jefferson University, 1015 Walnut Street, Philadelphia, PA 19107 USA; 3 College of Population Health, Thomas Jefferson University, 901 Walnut Street, Philadelphia, PA 19107 USA

**Keywords:** Drug checking, People who use drugs, FTIR, Drug overdose, Harm reduction

## Abstract

**Background:**

Adulterants, such as fentanyl and xylazine, among others, are present in a high percentage of the illicit drug supply, increasing the risk for overdose and other adverse health events among people who use drugs (PWUD). Point-of-care drug checking identifies components of a drug sample and delivers results consumers. To successfully meet the diverse needs of PWUD, more information is needed about the utility of drug checking, motivations for using services contextualized in broader comments on the drug supply, hypothesized actions to be taken after receiving drug checking results, and the ideal structure of a program.

**Methods:**

In December 2021, semi-structured interviews were conducted with 40 PWUD who were accessing harm reduction services in Philadelphia, PA. Participants were asked about opinions and preferences for a future drug checking program. Interviews were audio recorded, transcribed and coded using content analysis to identify themes.

**Results:**

Participants were primarily White (52.5%) and male (60%). Heroin/fentanyl was the most frequently reported drug used (72.5%, *n* = 29), followed by crack cocaine (60.0%, *n* = 24) and powder cocaine (47.5%, *n* = 19). Emerging themes from potential drug checking consumers included universal interest in using a drug checking program, intentions to change drug use actions based on drug checking results, deep concern about the unpredictability of the drug supply, engaging in multiple harm reduction practices, and concerns about privacy while accessing a service.

**Conclusions:**

We offer recommendations for sites considering point-of-care drug checking regarding staffing, safety, logistics, and cultural competency. Programs should leverage pre-existing relationships with organizations serving PWUD and hire people with lived experiences of drug use. They should work with local or state government to issue protections to people accessing drug checking programs and ensure the service is anonymous and that data collection is minimized to keep the program low-threshold. Programs will ideally operate in multiple locations and span “atmosphere” (e.g., from clinical to a drop-in culture), offer in-depth education to participants about results, engage with a community advisory board, and not partner with law enforcement.

## Background

The local drug supply in Philadelphia became increasingly unstable during and after the height of the COVID-19 pandemic [2, 14]. After over 1200 people died of a drug overdose in 2020, fatal overdoses rose to 1276 in 2021, the highest year on record [14]. A high percentage of the local drug supply has been contaminated with adulterants such as fentanyl and xylazine (a veterinary tranquilizer); drug checking results showed that, among 199 “dope” samples analyzed from Philadelphia in the first half of 2023, 99% contained fentanyl and 98% contained xylazine [24]. This has led to increased unintentional overdose among people who use drugs (PWUD) who are unknowingly ingesting drugs with these substances present [16] as well as an uptick in severe wounds and skin necrosis associated with xylazine use [1, 4]. Despite these harms, existing literature that examines the composition of the local drug supply is limited and may not be representative of the entirety of Philadelphia [24].

Drug checking is a harm reduction method that offers PWUD information about the contents of their drug. Fentanyl test strips (FTS), immunoassay paper strips used to detect fentanyl in different drug types and forms, are the most commonly known drug checking tool [8]. From its origins in the 1990s at festivals and nightclubs, this practice has been incorporated into other public health interventions to curb the opioid epidemic [6]. It serves the dual purpose of allowing PWUD to make informed decisions about their plans to use drugs while simultaneously creating a surveillance system of the drug supply [17]. Results are communicated back to PWUD to enhance informed decisions on how they use or alter the use of a drug. This is referred to as “point-of-care drug checking” [13]. This practice can also better establish the prevalence of harmful cutting agents in the local drug supply in Philadelphia by creating an aggregate database detailing drug testing and results.

Fourier-transform infrared spectroscopy (FTIR) is a characterization technique based on the amount of infrared radiation absorbed or emitted by the tested sample [13]. It can detect multiple substances (≥ 5% of the total volume) in a small drug sample (> 5 mg) and is particularly powerful when combined with FTS. FTIR devices are compact enough to apply to mobile services and scan samples within a few minutes, making it ideal for point-of-care services [5]. Used together, FTS can detect whether a sample has fentanyl, and the FTIR can expand on that detection to include other substances that comprise the drug sample [19]. Point-of-care drug checking programs using this technology exist in Canada, Australia, and Europe, among other places [9]. There are few established drug checking programs in the USA; however, more are in the early phases of development or implementation.

The information received by PWUD about the contents of drugs can reduce harm by changing drug use plans, such as smoking a sample contaminated with fentanyl instead of injecting it [26]. Knowing the composition of one’s drug can also potentially enhance desired effects (e.g., if testing confirms the drug contains what the consumer intends to use and shows no undesired contaminants), though research is needed to explore this area. PWUD can also communicate drug information to their peers and pressure illicit drug markets to create safer products [17]. This knowledge likely also benefits people who sell substances, as they often do not know the composition of their drugs and indicate an interest in keeping customers safe [26]. Potential benefits from these changes include decreased loss of life, reduced burden on the hospital care system, and linkages between regions to share drug composition trends [17].

Successful drug checking programs will meet the diverse needs of PWUD. Formative research conducted with PWUD has found that common concerns are centered on the criminalization and stigmatization associated with drug use and a fear of what exactly is in the drug supply [11]. Drug checking programs would have to overcome these barriers, with an emphasis on building rapport between providers and PWUD and providing non-judgmental harm reduction messaging. These services would potentially have impacts beyond the individual level, including improving the drug market, creating healthier communities, and supporting initiatives at the policy level [10, 22, 26, 27]. However, the specific perspectives of PWUD on the utility of drug checking services, motivations for using services contextualized in broader comments on the drug supply, hypothesized actions to be taken after receiving drug checking results, and the ideal structure of a program with concrete recommendations can be further explored. Therefore, this qualitative study aimed to assess the perceptions, barriers, and needs identified by potential service consumers to implement a drug checking program in Philadelphia.

## Methods

**Fig. 1:**
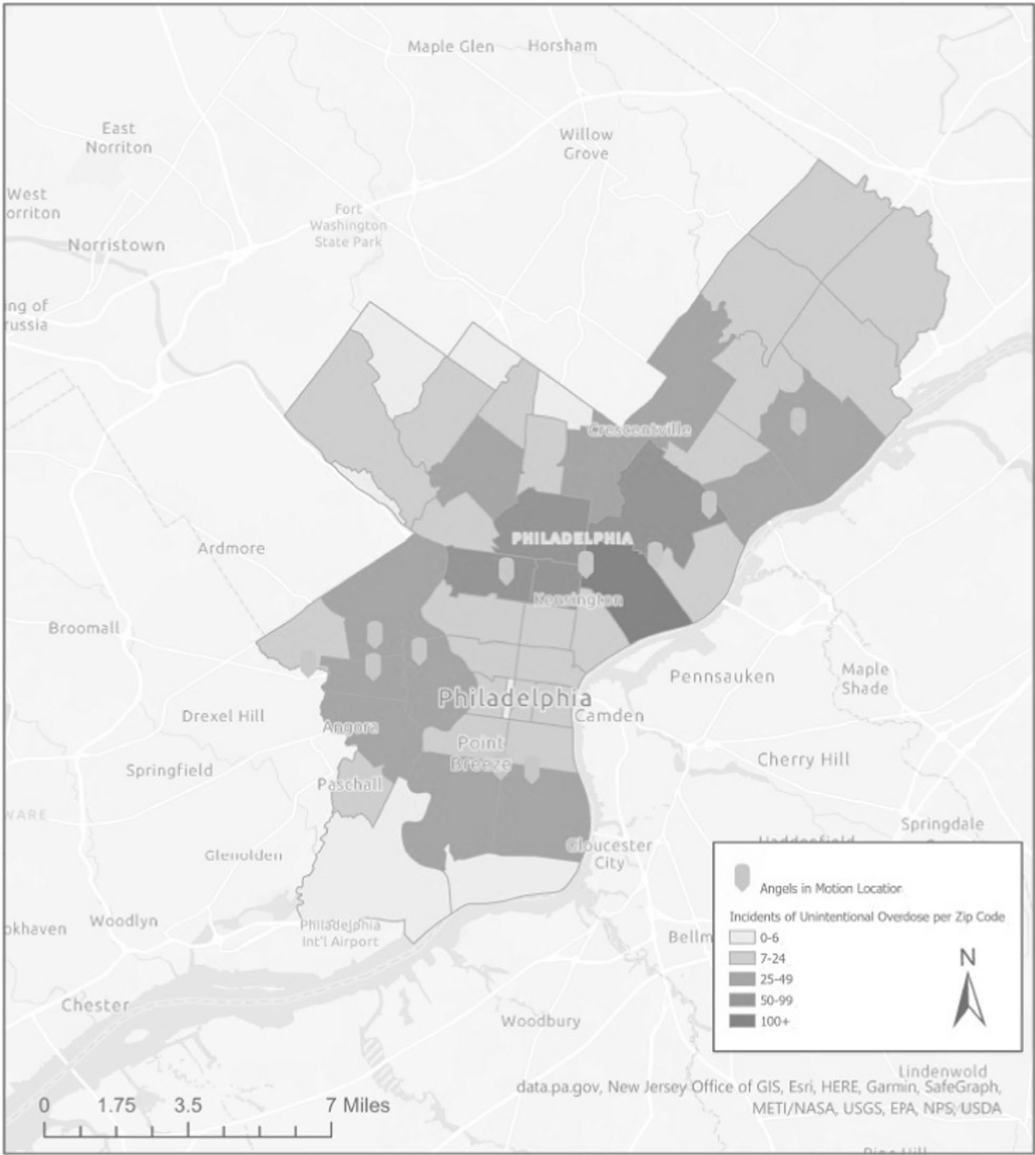
Angels in motion mobile sites and locations of fatal overdoses in Philadelphia, PA

This was a qualitative study using semi-structured interviews with PWUD. Methods are reported here using the Consolidated Criteria for Reporting Qualitative Studies (COREQ) [25]. Data collection occurred in December 2021 with individuals recruited at Angels in Motion (AIM) mobile sites. AIM was a harm reduction organization serving PWUD through eleven mobile sites in six neighborhoods in Philadelphia. Their services included syringe exchange, safer use supplies, and food services. AIM ceased operations in mid-2022. The program team interviewed at least one participant at every AIM site to capture a diverse range of voices from different communities across the city that are impacted by the overdose crisis (Fig. 1).

At the time of the interview, the legal status of drug checking in Pennsylvania was unclear, as FTIR equipment could be considered drug paraphernalia. The law was amended in November 2022 to clarify that drug checking is legal in the state [20]. The research team intentionally recruited with the goal of eliciting information from people representing a range of socio-demographic characteristics and different types of drug use. As recruitment progressed, the study team sampled specifically for certain types of drug use (e.g., crack cocaine) and for participants demographics (e.g., female, not White). Recruitment ceased upon saturation of interview content as agreed to by team members.

Those receiving services from AIM were approached and asked if they would like to participate in the interview. Participant inclusion criteria included: 18 years or older, English speaking, and current self-reported drug use. Participants were excluded if they could not provide consent as assessed by a team member during enrollment. Eligible participants were given details of the study and provided informed consent, then participated in a semi-structured interview. Participants were interviewed by authors MKR, a White female Assistant Professor with a PhD in Public Health, and TEC, a Latinx female Research Coordinator with an MPH, both of whom have qualitative experience engaging with PWUD. The interviewers did not have pre-existing relationships with study participants, nor did participants receive results of this study directly. Participants selected a semi-private location outside to be interviewed where they could not be overheard by AIM staff, other participants, or anyone else. Participants were asked to provide a pseudonym, represented here when reporting verbatim quotations, gender, race, and drugs used. All participants who were approached consented to an interview and all completed the full interview. A community advisory board comprised of people with lived experiences of drug use advised the study team on research protocols and interpretations of data. All study activities were approved by Thomas Jefferson University and the Philadelphia Department of Health Institutional Review Boards (IRBs).

Basic demographic information was collected using a pre-determined set of options, including gender, age, race, ethnicity, housing status, sources of income, frequency of drug use, types of drugs regularly used, overdose ever/last overdose, and the most common zip code of drug buying and use. Interviews were designed to be brief and not to interrupt the needs of participants accessing harm reduction services. It was adapted in part from Wallace et al. [26] and revised in consultation with research partners at the Philadelphia Department of Public Health. The interview guide was not piloted prior to data collection. The interview guide asked questions about opinions and preferences for a future drug checking program. This included perspectives on the current drug market (“How important is it to you to know what’s in your drugs?”), interest in a potential point-of-care drug checking program (“Do you feel like a drug checking service is something you would use?”), post-test actions (“What would you do with the information you get from the service?”), perspectives on the program design (“If you could design this program, what would it look like?”), concerns they had including privacy, police, and safety (“Just hearing about a program like this, what concerns come to mind?”), experiences with FTS (“Have you ever used an FTS? Is so, how often?”), and experience with AIM (“How long have you been working with the Angels? What services or supplies do you get from them?”). Interviews were audio-recorded and ranged from 4 to 17 min. Participants were offered $20 cash in remuneration for their time.

### Analysis

For data analysis, quantitative data were uploaded into REDCap and imported to SPSS for cleaning and univariate statistical analysis. All interviews were audio-recorded and transcribed verbatim through a HIPAA-compliant professional transcription company. Data were analyzed by four coders (authors MKR, TEC, EB, and MK) in NVivo11 software using a content analysis approach to identify salient themes [15]. The team developed a codebook through a combination of a priori codes (informed by the literature and interview guide) and line-by-line reading of subsections of the interview transcripts. Nearly 20% of interviews (*n* = 7) were coded by all team members to ensure codebook fidelity. Discrepancies were resolved in group discussions among the research team. Intercoder reliability was very high (kappa = 0.96).

## Results

A total of 40 potential service consumers participated in this study. Most participants were interviewed in the Kensington/Port Richmond area (40.0%, *n* = 16). Participants were primarily male (60%, *n* = 24), White (52.5%, *n* = 21), or Black (35%, *n* = 14), and the median age of participants was 39 years (Table 1). Most participants reported being undomiciled (62.5%, *n* = 25) and not having any source of income (60.0%, *n* = 24). Heroin/fentanyl was the most frequently reported currently used drug (72.5%, *n* = 29), followed by crack cocaine (60.0%, *n* = 24) and powder cocaine (47.5%, *n* = 19). Most participants reporting powder cocaine use also reported “speedballing”, mixing powder cocaine and heroin/fentanyl to inject together. Most participants (85%, *n* = 34) reported daily drug use and 60% (*n* = 24) reported ever experiencing an overdose, with 54.2% (*n* = 13) experiencing one within the last two years. The median number of overdoses ever experienced among participants was 4.5 (interquartile range 1.8). The 19,134 zip code in the Kensington neighborhood was the most frequent location reported for buying drugs (60.0%, *n* = 24) and using drugs (52.5%, *n* = 21).

**Table 1:** Characteristics of participants in qualitative interviews about drug checking in Philadelphia, PA (*N* = 40)

	*n* (%)
Age—mean (SD)	42.8 (12.0)
Gender identity	
Male	24 (60.0)
Female	16 (40.0)
Ethnicity	
Not Latino/Hispanic	35 (87.5)
Latino/Hispanic	5 (12.5)
Race	
White	20 (50.0)
African-American/Black	13 (32.5)
Native American	1 (2.5)
Other	6 (15.0)
Housing status	
Street, shelter, abandoned building	25 (62.5)
Own house or apartment	7 (17.5)
Single room occupancy	4 (10.0)
Family or friend’s house	4 (10.0)
Source of income^a^	
SSI/SSDI	7 (17.5)
Employed	3 (7.5)
Day labor	3 (7.5)
Panhandling	3 (7.5)
Retired	1 (2.5)
None	24 (60.0)
Types of drugs currently used^a^	
Heroin/fentanyl	29 (72.5)
Crack cocaine	24 (60.0)
Powder cocaine	19 (47.5)
Cannabis	11 (27.5)
Methamphetamine	6 (15.0)
Benzodiazepine	1(2.5)
Synthetic cannabinoid (“K2”)	1 (2.5)
Other^b^	4 (10.0)
Drug use patterns and overdose history	
Days of drug use per week-mean (SD)	6.4 (1.6)
Ever experienced an overdose	24 (60.0)
Number of lifetime overdoses—median (IQR)	4.5 (1, 8)
Last overdose was within the past 2 years	13 (54.2)
Neighborhood of interview	
Kensington/Port Richmond	16 (40.0)
Northeast Philadelphia	7 (17.5)
West Philadelphia	7 (17.5)
North Philadelphia	5 (12.5)
South Philadelphia	5 (12.5)
Zip code where you usually buy drugs	
Kensington/Port Richmond	24 (60.0)
Northeast Philadelphia	5 (12.5)
West Philadelphia	5 (12.5)
North Philadelphia	3 (7.5)
South Philadelphia	3 (7.5)
Zip code where you usually use drugs	
Kensington/Port Richmond	21 (52.5)
Northeast Philadelphia	7 (17.5)
West Philadelphia	5 (12.5)
North Philadelphia	4 (10.0)
South Philadelphia	3 (7.5)

^a^Results are not mutually exclusive and will not sum to 100%

^b^Participants reported synthetic cathinones, “D”, tranquilizers, and alcohol in the other category

Emerging themes from potential drug checking consumers included history of drug use, current risk mitigation strategies in an opaque drug market, interest in using a drug checking program, thoughts on potential drug checking consumers, how access to such a program might impact drug use, and ideal drug checking program structure. Verbatim quotes are presented here with the participant-select pseudonym, gender, race, and regularly used drugs.

### Risk mitigation strategies

Participants took several precautions when using drugs in the absence of current access to drug checking with an FTIR. Methods to reduce risk from a contaminated drug included waiting to use a drug until seeing the effect it had on other people, buying from the same person, using visual assessments to make an educated guess about substance composition, altering the drug in some way to see what color it became, or noting its smell. Among these, the most frequent protective measure used was going to the same person or place to buy drugs. One person mentioned carrying naloxone in the event of an overdose. One person talked about how emerging wounds changed his risk mitigation practices, stating:

That’s why my legs are all messed up now. Because it was – what the hell was the stamp, this shit called [stamp name], way on Franklin side. And I noticed that everyone that was using it, it looked like it just started eating your flesh away. And that’s what happened to my legs. And I’m still going through that now. So, I always wonder. And then they do samples, when they give out the free shit, I won’t even do mine until I see how it affects everybody else because I done had some bad experiences. – Derrick (male; Black; heroin/fentanyl, powder cocaine)

Out of 40 respondents, 24 people said they had used FTS. Among these, 22 reported current heroin/fentanyl use. Three participants used FTS daily, seven people used them when buying from a new person or using a new batch of drugs, four people used them when they had a concern about drug consistency, and three people used them out of curiosity. Barriers to participants using FTS included not knowing how to use them, trusting where they purchased drugs, and being afraid to know the result.

### Interest in drug checking

When asked how often they thought about what was in their drugs, many responded that they “always” or “often” thought about it, though twelve people said they did not think about it at all. These participants said they did not think about contamination or that even if they felt curious it ultimately would not matter what was in their drugs since they would use them regardless. Participant responses emphasized the complexity and difficulty PWUD face when interacting with the Philadelphia drug supply. Participants indicated a belief that the entire drug supply was saturated with fentanyl. This was often expressed through statements about fentanyl in the cocaine supply. None mentioned a concern of fentanyl in combination with cannabis. Aardvark (male; Black; heroin/fentanyl, crack cocaine, “D”) discussed the contamination of the local supply of crack cocaine and powder cocaine and said, “[…] they’re putting fentanyl pretty much in everything.” Some people were concerned about fentanyl not being present as they believed this meant that the drug would contain other unwanted adulterants with worse effects than fentanyl. Seventeen participants (42.5%) noted that certain adulterants/diluents/cuts of drugs led to adverse outcomes not associated with overdose. Akbar (male; Black/White; powder cocaine, methamphetamine, heroin/fentanyl) discussed what came to mind when buying drugs and said, “What the cut of the drug is because that leads to open wounds, lesions, things of that nature”. A minority of participants expressed a preference for fentanyl when buying “dope”.

Some people indicated that they did not like their lack of control over the drugs they bought. They often made statements which indicated resignation about what they consumed; however, these were usually followed by broader observations about the drug supply. This included micro-level factors such as profit incentives from sellers and the ubiquity of fentanyl. Participants frequently commented on xylazine as a common drug contaminant. Seven people mentioned xylazine or tranq by name, while others referenced “krokodil” or a “flesh-eating drug” that may have actually been xylazine. One participant discussed interest in drug checking due to the state of the current drug supply, saying:

I do get curious, especially like certain times where something – you can tell something’s off and you’re just like, what is – you know, you just sort of want to know what it is, even if you’re still going to end up using it, which sounds stupid, but – you know? … It’s pretty important. I would say that’s at about a ten if you’re on a scale just because, again, you don’t know at all. You have no idea. They could be putting anything in it. And what if you have a medicine allergy that they use that to cut – whatever. You know, stuff like that. Like you could literally die. I mean you could die anyway, but there’s things that add risks. It’s not very available. Because there’s fentanyl test strips and stuff, but other than that you can’t really find out, so I think it would be really great to have more opportunity for it because it is really important. – Sally (female; White; heroin/fentanyl, powder cocaine)

Overall, participants found drug checking appealing due to the ability to exert control over an unregulated drug market to safeguard their health and safety. All participants indicated during at least one point in the interview that they were interested in using a drug checking service, though some were more enthusiastic than others. Some indicated during the interview that they would not use a service but later said they would use it for a specific reason, for example if they decided to go into a substance use disorder treatment program. Common reasons listed for wanting to use a drug checking service included wanting to find heroin/fentanyl with the fewest numbers of cuts (*n* = 21, 52.5%), out of curiosity or to be equipped with knowledge (*n* = 18, 45.0%), to know that the drug would provide the desired use experience they were seeking (*n* = 13, 32.5%), when going to a new seller or getting a new “stamp” (different branding of product purchased) (*n* = 4, 10.0%), or when purchasing from a location perceived to have unsafe drugs (e.g., Kensington, a neighborhood in Philadelphia associated with public drug consumption) (*n* = 1, 2.5%).

One unanticipated finding from five interviews spoke to the inadequacy of current protocols for people seeking medically-managed withdrawal (“detox”). This was often attributed to the presence of xylazine in the drug supply. Ashley (female; White; powder cocaine, crack cocaine, heroin/fentanyl, synthetic cathinones [“bath salts”]) discussed the presence of xylazine (“tranq”) in the drugs she used and said, “I go to a [buprenorphine] clinic and I talk to this doctor there and she said there’s really nothing that you can do for detox with the tranq. So ideally, I don’t like the effects that tranq has, I don’t like what it does to me, and I don’t like the idea of not being able to have something to help with detox”. Another unanticipated finding was the utility of drug checking to anticipate results from a future urine drug screen.

### Thoughts on potential drug checking consumers

Participants were asked who should use a drug checking service. Interviewees believed that program participation would be widespread. The majority felt that most PWUD would want to use the service. One person believed that only people that have overdosed in the past would like to use the service, as they would then be more concerned about the content of their drugs.

Overall, participants believed that the program could have a wide-reaching client base and that people who did not use drugs could also utilize the program. These other groups included family, parents, or other concerned individuals that did not use drugs themselves. One participant discussing potential consumers of the program said:

I believe not only addicts but other people who aren’t addicts [would use a drug checking program], people that know about the addiction, and people that may have either suffered from abuse of addiction or people or family members or friends that have also suffered from it. – William (male; White; heroin/fentanyl, powder cocaine, crack cocaine, methamphetamine)

### Prospective post-test actions

People named a range of actions they would take after using a drug checking service. Responses varied among participants who used heroin/fentanyl compared to those who did not. Among those with heroin/fentanyl use (*n* = 29), most said they would still use a drug with unfavorable test results. One person said he would stop using drugs entirely, and another said he would cut back on use. A few people said they would not use the drug, though one person noted that it depended on whether she was “dope sick”. Some interviewees said they would use standard harm reduction recommendations if test results returned unfavorably (as in the case of xylazine contamination of fentanyl/heroin) by using less of that drug. Two people said they would insufflate or smoke their drug instead of injecting, and a few people noted that results might influence long-term changes in their drug practices. Derrick (male; Black; powder cocaine, heroin/fentanyl) discussed how a drug checking service might change his drug use and said, “I know it would slow me down and probably make me realize that this ain’t for me. Because like certain things have a tendency to scare me”.

Participants who did not use opioids (*n* = 11) indicated different hypothetical actions they would take after receiving drug checking results. Five out of 11 said that if fentanyl were present in their non-opioid drug, they would stop using drugs entirely. Almost all indicated they would not buy from the same person or place again. Only one person said he would still smoke a drug that tested positive for fentanyl.

Across all types of drug use, participants frequently indicated that unfavorable drug checking results would lead them to tell other people what they had learned and buy from a different seller. A few said they would tell the drug sellers themselves about drug checking results, such as Sally (female; White; crack cocaine, heroin/fentanyl):

Probably go back to whoever I bought it from. And you know, like, come on. You know? Because they are – sometimes with dealers and stuff, they’re still people, so a lot of times they will understand and be real with you and like, all right, cool, you can have your money back or when the next batch comes in, I’ll give you something. They do do that. So I would call them on that, even if it just is so that it can be avoided the next time. You know what I mean?

### Drug checking concerns

When asked in general terms about concerns associated with a drug checking program, participants initially expressed no worries about a program and were more encouraged by its potential to help them. However, some concerns arose when explicitly asked about legal/police issues, privacy, or safety issues.

Generally, participants were not concerned about a service having unclear legal status but were concerned about the presence and involvement of the police. Nine out of 40 participants discussed specific worries about policing and drug checking. These participants were five people of color (3 Black and 2 Hispanic/Latino participants) and four White participants). Universally, those who did express legal concerns said that police should not be involved in any way, including running the program or gathering information from the program. Police involvement would lead them to not participate. A few participants expressed concerns that the drug checking program would serve as a hotspot for police presence, where officers would make mass arrests for drug possession. Because of this, a few participants said they would prefer it if the service was “low key” and not advertised throughout the city. Allison (female; White; crack cocaine, heroin/fentanyl, cannabis) expanded on this and said, “Of course, it’s gonna end up getting caught on and known, but as long as it’s not I guess where the cops are coming all the time and people want to try and catch us as we walk away, because they know we have shit on us that we’re trying to get tested.”

A few participants said they would be more comfortable using the service if it were explicitly legal or had reassurances from the city that they would not be prosecuted for participating. One interviewee was concerned that participating in a program would make her ineligible for other social services from the city. Another with no legal concerns said that police would likely support such a program as it would reduce drug overdoses.

Half of the people who answered questions about privacy regarding a drug checking service indicated no concerns. Among those who were concerned, confidentiality, discretion, and anonymity came up repeatedly. A few participants said they would prefer a service off the streets and private so as not to be seen by others, including children, friends, or family. These participants tended to use AIM sites outside of Kensington. Most concerned participants brought up issues about their personal information being compromised and preferred a system that preserved anonymity. Some ideas to achieve this included using code names or signing a confidentiality form. They were also concerned about a program that might collect and share personal and medical information and would be discouraged from using a program that collected such information.

One participant discussing what would make him comfortable using the program said:

Just show up, real in and out the door, no names. You know what I mean? People, they like the anonymity of things. They don’t really like per se … People don’t want to go there and think they’re gonna get arrested or something. Oh, yeah, but you know what I mean? So kind of like that, just knowing that you’re not gonna get in trouble for that - Josh (male, White, heroin/fentanyl, powder cocaine, crack cocaine, cannabis)

Seven of the ten people who answered questions regarding safety were not concerned. Of those concerned, all were men. One said he was worried about people taking advantage of those who used the program to rob them. Another mentioned that he might encounter someone with whom he had issues in the past and would be best served if the program was in a controlled environment. One participant proposed that security guards would make him feel safer. Interviews were analyzed to determine whether women had greater safety concerns than men and no differences were detected.

### Ideal characteristics of a drug checking program

Participants indicated that several factors could lead to a good or bad experience when interacting with a proposed drug checking program. Many had difficulty answering questions about how a drug checking program should be structured as the service was a new concept to them. Important attributes regarded trust in the organization operating a drug checking service and thoughts on locations, dates, and times for a program.

Trust in staff was critical in willingness to engage in a drug checking program. Participants wanted the staff to be empathetic, honest, judgment-free, and welcoming. The participants requested that the team be upfront with them and treat PWUD as human beings. Many indicated that sterile or formal staff members would be alienating.

Eight people said that staff with lived experience would be necessary for the program. One of the participants who wanted someone with lived experience felt that those individuals should have ample time in recovery to minimize “triggers” and a subsequent return to use. Three participants said they would trust medical personnel to run the program; two of those respondents said they would prefer the medical personnel to have lived experience. Two participants mentioned other non-profit organizations they would trust to operate a drug checking program. One participant discussing his preference for staff with lived experience said:

You would have to have people working the facility that has been through the experience. Because a person that has never done it, they can’t really grasp the concept of what’s going on with us. And that’s what really irritates me. Because I’m like how can you tell me about something you never done? Just because somebody told you about it, or you read about it, don’t mean you know about it until you experience it. – Derrick (male; Black; heroin/fentanyl, powder cocaine)

All participants said that it would be necessary to expand hours and days of operations at the site at which they were being interviewed, including having multiple buses if the service were mobile. However, there was a lack of consensus about ideal program days and location. Nine participants said that the program would have to be operating 24/7 for people to think to use the drug checking program regularly. One person reasoned that this schedule would ensure no one who wanted to check their drugs would miss the opportunity. Four participants said regular business hours would be ideal, and eight participants described a variety of more extended hours and days of the week than those present at the interview location. One person suggested splitting the times available at one location into a morning and a night shift. Another participant suggested running the program on a smaller scale (similar to a food cart) that could be easier to run in multiple locations. Four participants said that the drug checking site should be located in a brick-and-mortar location. These location ideas included working with another local harm reduction organization or partnering with businesses in the Kensington area. Most participants said that the site should be in Kensington since it was where they bought drugs and has a high density of drug use. While most people identified Kensington, two participants said it would be better in locations with high drug use but fewer services available, such as Southwest Philadelphia. One participant describing his preference for a Kensington location said:

This program should be something that is assistive. It doesn’t come across as controlling, just something that is here to make sure that whatever we’re utilizing we’re aware of it. It’s not here to aid us or to make us better drug users but to just let us know – aware of what we’re putting into our bodies. The location would have to be ground zero, because that’s where everything usually is transpiring, taking place. Ground zero is here in Kensington. – Akbar (male; White/Black; heroin/fentanyl, powder cocaine, methamphetamine)

The program could also combine with other treatment services to provide more comprehensive care. Six people felt that other supportive resources should be available with drug checking, including items for people in need like food and clothing. For example, Linda (female; Black; cannabis) said: “We need something like a clothing class for people who don’t have clothes”.

Participants noted other program features that could influence participant uptake and continued use of the program. A consistently important aspect was confidence that test results were accurate. One participant said that if there were general information about the drug supply available through aggregating all drug checking results, it would be helpful for PWUD that did not want to bring their drugs but wanted general information on the drug supply. Participants widely wanted the timing of testing to be prompt, with some saying that waiting over 10–15 min for results would be too long. One participant was adamant that all steps of the drug testing process be visible, as many PWUD would not trust a process that took a drug out of their possession. If the program location were too busy or filled with “prying eyes,” they would be less likely to have a positive experience. Participants were concerned about the amount of drug used for the testing but were comforted when researchers explained that drug checking personnel would need less than the size of a grain of rice. Three people stated they would only be interested in point-of-care testing. They were less interested in different models in which results are delayed since the process requires mailing some of their drugs and returning later for a result. Multiple participants said that the program should take participant feedback into account for longitudinal growth through a community advisory board.

## Discussion

In this qualitative study of potential consumers of a point-of-care drug checking program in Philadelphia, support and interest were unanimous. However, some initially said they would not use such a program, which echoes survey results from people using opioids in a fentanyl-saturated market [23]. However, all participants who initially voiced disinterest later indicated they would use a drug checking service. Participants indicated that results would often influence their decision about whether and how to use a drug that had been tested. Evaluations of drug checking programs show a change in intended or actual drug use after utilizing a drug checking service, though the focus of most studies have been festival attendees or limited to evaluating fentanyl test strip use [18].

Participants already engaged in several harm reduction strategies with their drug use and 60% had experience using FTS, indicating a broad interest in drug checking. This may indicate that those who receive point-of-care drug checking results may change how they use a drug (e.g., deciding not to use it, to use less, to administer in a different manner) or the environment of their drug use (e.g., using with a trusted person, carrying naloxone). It is key to recognize that not all participants can or will change an intended course of action due to test results. This may be due to having confirmed that the desired substance is present or may represent the reality that buying more of a drug is not economically or logistically feasible. This underscores the importance of pairing harm reduction messaging with test results.

Participants were curious about the composition of the drug supply and had previously made attempts to learn what was in their drugs. Participants indicated that the program would also be helpful for people who did not use drugs, such as family or friends. This supports previous findings from Wallace et al. [26, 27] that drug checking has the potential to have not only individual-level impacts, but can influence drug-related outcomes at the community level, as well. This may be due to word-of-mouth and distribution of aggregate drug checking results.

Participants named some specific concerns and operational considerations that were important to them. Similar to findings from a recent scoping review and two formative studies, participants in this study named convenient locations and hours of operation, along with privacy, as key considerations for a person-centered intervention [11, 27]. The desire to have a service operated through a trusted harm reduction partner, especially if staffed by people with lived and living experiences of drug use, is noted in some qualitative studies with potential service consumers in Scotland and service providers in the United States [7, 12].

Our findings are important as they can inform the structure of drug checking programs elsewhere from the perspectives of people who will be using the service. Based on participant interviews, we offer a number of considerations and recommendations for sites considering point-of-care drug checking, specifically for people with polysubstance use (see Table 2). Addressing elements from Table 2 will decrease the risk that a new point-of-care drug checking program will not meet the needs of more vulnerable participants (e.g., people on probation since incarceration is a risk factor for overdose, undomiciled people) [3, 21].

**Table 2:** Recommendations and considerations for developing drug checking programs

Staffing	Leverage the relationships already built between harm reduction agencies and communities of PWUD. Agencies with established trust and experience should operate drug checking programs. This is an important determinant of the success of a program, especially for clients who may feel ambivalent about drug checking. Operating programs successfully will require long-established trust within the community due to needing to hand over a sample of an illicit drug to staff to complete FTIR analysis. It is also helpful to have this trust established among participants who may be skeptical about drug checking technology or the results being produced
	Hire people with lived experience of drug use and/or people with clinical medical experience
Safety	Work with leadership at the city, county, or state level to anticipate potential legal challenges to drug checking work. When state laws do not allow drug checking, consider working with local government to issue protections (e.g., a Mayoral Order) to allow drug checking locally
	Information given to participants should not be able to be linked back to individuals. If necessary, participants in the program should be identified through the use of a unique identifier similar to the one they use for a syringe exchange program yet be separate. The use of a unique identifier should be explored to allow participants to access results at a later date, track engagement with the program, and for other program evaluation activities
Logistics	When feasible, operate multiple drug checking sites. Consider ways to make sites responsive to the diverse needs of PWUD to offer a spectrum of sites. Those preferring a more clinical environment could access programs with clinicians on staff with or without lived experience of drug use to answer questions about medical implications of drug components. Others who want on-demand access to a program staffed by people with lived experience could visit a “brick and mortar” site and interact directly with peers
	Situate a drug checking service within a larger suite of harm reduction services. The service should offer fentanyl test strips, naloxone, wound care, and safer injection equipment. It is critical that linkages to detoxification and other drug treatment programs be available given the high number of people who indicated they would want to cease drug use upon receiving results
	Participants should be offered printed materials after testing, but many will prefer to have a verbal conversation about results. Handouts should include terms of service stating what the drug checking program can and cannot tell them and a list of standard precautions to take when using drugs. A pre-determined fact sheet for each of the most common adulterants/diluents (e.g., xylazine, levamisole) that contains information on potential health consequences of use and harm reduction options can be given out with this information when those substances are detected
	Aggregate all test results and publish them as community reports using both health alerts during “surge” events and as general information for PWUD
Cultural competency	Most participants engaged in polysubstance use. Programs should expect a diverse range of drugs to be brought in and should be familiar with all illicit drugs and their most common adulterants and diluents
	Establish and meaningfully engage a community advisory board to provide program feedback and make recommendations for changes
	Consider the use of a participant fact sheet about the program prior to implementation to address and allay concerns related to the transparency of the process, amount of drug needed for the test, etc
	To build trust, programs may be more likely to succeed if they are not viewed as partnering with police. There should be legal protections for people using the service and there should be no police presence while the service is operating

### Limitations

We talked to people in one metropolitan area already accessing harm reduction services so they may have been more favorably disposed to additional harm reduction programs. While we intentionally sampled across geographic regions of Philadelphia, 40% of the sample was recruited from the Kensington neighborhood, potentially leading to overrepresenting themes most salient to PWUD in that neighborhood. The sample of PWUD may not represent the broader community of PWUD in Philadelphia; they may have different needs and preferences. Interviews were brief and conducted in semi-private locations. Longer interviews in a more controlled environment may have elicited different information. The concept of drug checking was new to participants; therefore, recommendations made may not work for participants if implemented as originally described.

## Conclusions

In this qualitative study, PWUD identified ideal characteristics of a hypothetical drug checking program. Themes included staffing, safety, logistics, and cultural competency. We assert that considering concerns raised and preferences named within each theme are one step of many to ensure that point-of-care drug checking programs are safe and effective for participants.

## Data Availability

Due to the topical and methodological nature of this research, participants of this study did not agree for their data to be shared publicly, so supporting data are not available.

## Abbreviations

AIM: Angels in motion
FTIR: Fourier-transform infrared spectroscopy
FTS: Fentanyl test strips
PWUD: People who use drugs
